# National estimates of poisoning events related to liquid nicotine in young children treated in US hospital emergency departments, 2013–2017

**DOI:** 10.1186/s40621-019-0188-9

**Published:** 2019-04-01

**Authors:** Joanne T. Chang, Baoguang Wang, Cindy M. Chang, Bridget K. Ambrose

**Affiliations:** Center for Tobacco Products, U.S. Food and Drug Administration, 10903 New Hampshire Avenue, Silver Spring, MD 20993 USA

**Keywords:** ENDS, E-cigarettes, NEISS, CPSC, E-liquid, Nicotine poisoning

## Abstract

**Background:**

An estimated 2 million youth (in 2017) and 7.9 million adults (in 2015) reported currently using electronic nicotine delivery systems (ENDS). Reports of poisoning events related to liquid nicotine (e-liquids) in ENDS have been on the rise, but current, nationally-representative estimates of hospital-treated poisoning cases related to e-liquid nicotine exposure in the United States (US) are lacking.

**Findings:**

We used National Electronic Injury Surveillance System (NEISS) data from 2013 to 2017 to calculate national estimates with 95% confidence intervals (CIs) of poisoning incidents related to e-liquid nicotine exposure. From 2013 to 2017, an estimated 4745 poisoning cases related to e-liquids among children under age five were treated in US hospital emergency departments; the number of cases increased from 181 (95% CI: 0–369) in 2013 to 1736 (95% CI, 871–2602) in 2015 and then decreased to 411 (95% CI, 84–738) in 2017. Most of the cases were treated and released; 4.1% were admitted to the hospital. The most common route of exposure was through ingestion (96.9%), and 2.6% of the cases were through dermal exposure. The highest amounts of e-liquids or nicotine ingested were 118.2mL, 1 bottle, and 100 mg, and the most common symptoms (63.6%) related to nicotine poisoning were nausea and vomiting.

**Conclusions:**

This study provides national estimates of poisoning cases associated with nicotine exposure from e-liquids among children under age five. Findings on e-liquid volume or nicotine dose, when available, provide important insights into exposures associated with toxicity in children. Since NEISS data do not include product codes specific to ENDS or provide information on poisoning severity, we used general keywords to capture these events, which might underestimate the population burden. Information from this study may complement efforts, such as public education, to prevent unintended exposure to nicotine in e-liquids among children.

**Electronic supplementary material:**

The online version of this article (10.1186/s40621-019-0188-9) contains supplementary material, which is available to authorized users.

## Background

Electronic nicotine delivery systems (ENDS) and electronic cigarettes (e-cigarettes) are battery-operated devices designed to deliver nicotine-containing liquids (e-liquids) and other additives to users in an aerosol form (US Food and Drug Administration 2018). In 2017, an estimated 1,730,000 high school students (11.7%) and 390,000 middle school students (3.3%) in the United States (US) reported using ENDS in the past 30 days (Wang et al. 2018) and in 2015, an estimated 7.9 million of US adults (3.5%) were current ENDS users (Phillips et al. 2017). With the high prevalence of ENDS use, potential ENDS safety concerns have been raised, including burn events related to battery explosions and poison events related to e-liquid nicotine exposure (Chatham-Stephens et al. 2016; Govindarajan et al. 2018; Kamboj et al. 2016; Vakkalanka et al. 2014; Wang and Rostron 2017; Corey et al. 2018; Rossheim et al. 2018).

Prior studies have characterized ENDS-related adverse events using data from the National Poison Data System (NPDS) (Chatham-Stephens et al. 2016; Govindarajan et al. 2018; Kamboj et al. 2016; Wang and Rostron 2017); One study used NPDS data from 2012 to 2017 to estimate the annual liquid nicotine exposure rate in US children under age six (Govindarajan et al. 2018). However, these studies have some limitations. NPDS is a data repository of information from telephone calls to poison control centers (PCCs) concerning poisoning exposures. PCCs’ primary goal is to provide professional advice on managing the poisoning exposures rather than provide treatment. Serious cases may bypass calls to PCCs and have gone directly to hospital emergency departments. Therefore, the majority of the ENDS-related poisoning exposure cases tracked by PCCs had minor effects (minimally bothersome signs or symptoms); less than 2% had moderate effects (more pronounced and prolonged symptoms, but not life-threatening); and less than 1% developed life-threatening symptoms (Chatham-Stephens et al. 2016; Govindarajan et al. 2018; Kamboj et al. 2016; Wang and Rostron 2017).

In this study, we analyze data from the National Electronic Injury Surveillance System (NEISS) to estimate the population burden of e-liquid poisoning events among young children treated in US hospital emergency departments (EDs) from 2013 to 2017. This is the first study to provide population estimates of poisoning events related to e-liquids in children under age five. Where available, we provide information on nicotine dose or concentration and e-liquid volume of exposure associated with these events.

## Methods

Maintained by the US Consumer Product Safety Commission (CPSC), NEISS collects data from approximately 100 US hospitals with at least six beds that provide 24-h emergency services (Hefflin et al. 2004; Schroeder and Adlt 2004; US Consumer Product Safety Commission 2018) that are selected as a nationally-representative probability sample of the approximately 5000 US hospital EDs, with the goal of providing national estimates of the number of injuries treated in EDs (US Consumer Product Safety Commission 2018). These hospitals are grouped into five strata, four representing different hospital sizes measured by number of annual ED visits, and the fifth representing EDs from children’s hospitals (Hefflin et al. 2004; Schroeder and Ault, 2001). Each case in the NEISS database includes information on demographics, diagnosis, disposition, affected body part, product code, and a 142-character text narrative of the incident. For tobacco-related poisonings, data are collected only for children under age five (US Consumer Product Safety Commission, 2018a, b). In this study, we analyzed the data on poisoning events related to e-liquid exposures from 2013 to 2017 for children under age five, which is consistent with the timeframe used in the 2012–2017 NPDS analysis by Govindarajan et al. (2018). Because only two cases were observed in the 2012 NEISS data, that year was excluded from the current analysis.

To identify poisoning events related to ENDS and e-liquids, we first conducted a text search of the narratives, including keywords such as “cig”, “liquid”, “nicotine”, “juice”, “e-cig”, “ENDS”, “e-liquids”, “eliquid”, “e-cigarettes”, “vapor”, “vape”, “e-hookah”, “hookah-pen”, “e-pipe”, “juul”, “jew”, and “pods.” To identify poisoning-specific events, diagnosis code 68 (“Poisoning”) was used (US Consumer Product Safety Commission, 2017a, b). NEISS defines poisoning as a swallowed liquid, soluble chemical or medication or inhaled vapors, fumes or gases. Since product codes were not specific to ENDS, we reviewed events with keywords related to ENDS and poisoning, and excluded events not related to e-liquid poisoning.

Since the diagnosis code is not specific to e-liquid poisoning events, we reviewed the text narrative involving e-liquid cases and identified routes of exposure (ingestion, dermal, or ocular). We identified cases related to ingestion using keywords such as “drink”, “drank”, “ingest”, “chew”, “sip”, “swallow”, “suck”, “mouth”, and “tongue”; cases related to dermal exposure using keywords such as “skin” and “dermal”; and cases related to ocular exposure using keywords such as “eye” and “ocular”. We extracted cases with nicotine volume or concentration of e-liquid information using keywords such as “bottle”, “cc”, “drops”, “mg”, “mL”, “oz”, “sip”, “tsp”, “%”, and “mg/mL”, and obtained cases with information on symptoms related to e-liquid exposure using keywords such as “eye redness”, “threw up”, “nausea”, “vomit”, “oral”, “emesis”, “diarrhea”, “crying”, “sleepy”, “coma”, “seizure”, “syncope”, and “cough”. Lastly, two reviewers manually reviewed all of the extracted event narratives independently, and excluded events related to cigarette poisoning and events not related to e-liquid exposure. All events were identified using the “FIND” procedure in SAS program (version 9.4; SAS Institute, Cary, NC).

### Statistical analysis

We calculated weighted population estimates and 95% confidence intervals (CIs) of poisoning events related to ENDS e-liquid nicotine exposure from 2013 to 2017, and estimated the weighted distribution of events by the following characteristics: age groups (younger than 2 years and 2–4 years), sex (males and females), race (White, Black, Other/mixed Race, and unknown), route of exposure (ingestion, dermal, other, and unknown), and disposition (treated and admitted, treated and released, and left without being seen). All analyses were conducted using the SAS program and using PROC SURVEY to account for the complex designs and sample weights. We converted all units (CC, drops, tsp., sip, and oz) into milliliters (mL) and calculated the unweighted frequency of events and the range (minimum, maximum, mean and median) of e-liquid volume. We also calculated the unweighted frequency of events for the top five symptoms reported. CPSC considers the estimates to be unstable if (1) the estimate is less than 1200; (2) the number of records used is less than 20; or (3) the coefficient of variation (CV) is greater than 33% (US Consumer Product Safety Commission, 2017a, b).

Because only anonymized, publicly-available data were used, the study was considered exempt from human subjects committee review.

## Results

Between 2013 and 2017, 116 poisoning cases were identified in the NEISS data, representing a national estimate of 4745 (95% CI: 2894-6696) poisoning cases related to e-liquids among children under age 5 who were treated in US EDs. The number of poisoning cases increased from 181 (95% CI: 0–369) in 2013 to 1736 (95% CI: 871–2602) in 2015 and then decreased to 411 (95% CI: 84–738) in 2017 (Table 1). The majority of poisonings occurred in males (54.3%), Whites (58.2%), and those aged 2 years or younger (56.2%). While the majority of poisoning cases were treated and released from the hospital, 4.1% were admitted. The most common route of exposure was through ingestion (96.9%), and 2.6% of the cases were through dermal exposure. We found 46 out of a total of 116 unweighted cases (39%) with case narratives containing e-liquid volume, nicotine dose or nicotine concentration information. Before unit conversion, 59% of these cases were reported in bottle(s) and 25% were reported in mL (Additional file 1: Table S1). Other cases (15%) reported other measurements such as drops, CC, tsp., sip and oz., and these were converted to “mL”. Two cases reported nicotine concentrations as 18% and 12 mg/mL. After unit conversion, the highest amounts associated with reported exposures were 118.2 mL of e-liquid, 1 bottle of e-liquid, and 100 mg of nicotine (Table 2). We also found 11 unweighted cases that reported information on symptoms (Additional file 1: Table S2), with the most common symptoms being vomiting and nausea (63.6%) (Table 3).

**Table 1:** Electronic nicotine delivery system (ENDS) e-liquid nicotine-related poisoning events in the United States (2013–2017)

Characteristics	2013		2014		2015		2016		2017		2013–2017	
	Unweighted N	N (%)	Unweighted N	N (%)	Unweighted N	N (%)	Unweighted N	N (%)	Unweighted N	N (%)	Unweighted N	N (%)
All	13	181^b^	36	1000^b^	33	1736	25	1416	9	411^b^	116	4745
Age												
Less than 2 years old	6	121 (66.9)	18	462 (46.2)	15	782 (45.0)	17	979 (69.1)	6	322 (78.4)	62	2667 (56.2)
2–4 years	7	60 (33.1)	18	538 (53.8)	18	954 (55.0)	8	437 (30.9)	3	89 (21.6)	54	2078 (43.8)
Sex												
Males	7	59 (32.5)	21	477 (47.7)	19	1010 (58.2)	14	776 (54.8)	6	252 (61.3)	67	2574 (54.3)
Females	6	122 (67.5)	15	523 (52.3)	14	726 (41.8)	11	640 (45.2)	3	159 (38.7)	49	2171 (45.7)
Race												
White	9	130 (71.9)	20	465 (46.5)	18	1063 (61.2)	14	778 (54.9)	7	327 (79.6)	68	2763 (58.2)
Black	0	–	2	29 (2.9)	3	200 (11.5)	0	–	1	5 (1.2)	6	233 (4.9)
Other/mixed race	0	–	0	–	2	161 (9.3)	2	157 (11.1)	0	–	4	317 (6.7)
Unknown	4	51 (28.1)	14	507 (50.7)	10	313 (18.0)	9	482 (34.0)	1	80 (19.3)	38	1432 (30.2)
Disposition												
Treated and admitted to a hospital	1	7 (3.7)	4	51 (5.1)	4	52 (3.0)	–	–	2	84 (20.4)	11	194 (4.1)
Treated and released	11	160 (88.1)	31	943 (94.3)	29	1684 (97.0)	25	1416 (100)	7	327 (79.6)	103	4530 (95.5)
Left without being seen	1	15 (8.2)	1	6 (0.57)	–	–	–	–	–	–	2	21 (0.43)
Route of exposure												
Ingestion	13	181 (100)	31	852 (85.2)	33	1736 (100)	25	1416 (100)	9	411 (100)	111	4597 (96.9)
Dermal	0	–	3	43 (4.3)	0	0	0	0	0	0	3	858 (2.6)
Other^a^	0	–	1	6 (0.57)	0	0	0	0	0	0	1	6 (0.12)
Unknown	0	–	1	100 (10.0)	0	0	0	0	0	0	1	100 (2.1)

^a^Case with more than one route of exposure, both ingestion and occular

Unweighted N = unweighted counts

N = Estimated Frequency, produced by applying statistical weights provided by CPSC to the unweighted counts

% = distribution by specific characteristics

^b^CPSC considers a national estimate unstable and potentially unreliable when the weighted estimate is less than 1200

95% CI: 95% Confidence Interval

**Table 2:** ENDS e-liquid nicotine-related poisoning cases (2013–2017) with information on nicotine dose (mg) or e-liquid volume (mL or bottle)

Unit of measurement	Sample size (%)^b^	Dose range			
		Minimum	Maximum	Median	Mean
mg	6 (14%)	1.8	100	12	3
mL^a^	19 (43%)	0.2	118.3	3.5	16.8
bottle	26 (59%)	0.5	1	1	0.875

^a^8 out of 19 cases with volume information on “drops, c.c., tsp., sip and oz” were converted to “mL”

^b^5 out of 46 cases have more than one unit of measurements

**Table 3:** Reported symptoms in ENDS e-liquid nicotine related poisoning cases (2013–2017)

Reported Symptoms^a^	Sample Size (%)
Vomiting, nausea, emesis	7 (63.6%)
Crying, eye redness	2 (18.2%)
Cough	1 (9.1%)
Sleepy	1 (9.1%)
Oral cyanosis/Unresponsive^b^	1 (9.1%)

^a^11 out of 116 total cases (9.5%) have information on symptoms

^b^cyanosis: blue-colored skin caused by too little oxygen in the blood

One case has multiple symptoms

## Discussion

We analyzed nationally-representative data to estimate the population burden of poisoning events among young children associated with nicotine exposure to e-liquids in ENDS products from 2013 to 2017. An estimated 4745 cases of poisoning injuries presented to EDs in the US. Previous studies investigating poisoning exposures to e-liquid nicotine used data from NPDS or hospital case reports, and were unable to calculate the population weighted estimates of poisoning cases related to e-liquid nicotine. The national estimates of poisoning events related to e-liquids among children under age five peaked in 2015 and dropped afterward. These results reflect similar patterns shown in the study by Govindarajan et al., which reported an annual exposure rate increase from 0.7/100,000 in 2012 to 10.4/100,000 in 2015 and decrease to 8.3/100,000 in 2016 (Govindarajan et al. 2018).

Many states were not required to have child-resistant packaging for liquid nicotine (Frey and Tilburg 2016) until the Child Nicotine Poisoning Prevention Act (CNPPA) of 2015 became effective in 2016 in accordance with CPSC standards. Although the decrease in poisoning cases related to e-liquid nicotine in children could be attributed in part to increased awareness of state and federal legislations, there were still an estimated 411 cases in 2017. Furthermore, education programs designed to increase awareness of the potential harms of ENDS and the need to keep ENDS products out of reach of young children may help reduce and prevent ENDS-related adverse events in children.

Our results showed that almost all poisoning cases among young children occurred through ingestion. While less than 50% of case narratives contained information on nicotine dose, concentration, and volume of ingested e-liquids, we found that the highest reported amount of nicotine dose ingested was 100 mg. Case reports have shown that a lethal dose of nicotine in adults is 60 mg or less (30–60 mg) and 1 mg/kg in children (Gupta et al. 2014; Mayer 2014; Seo et al. 2016). However, scientific evidence for the human lethal dose estimates is difficult to obtain because this information can only be estimated postmortem. Information on e-liquid volume and nicotine concentration provides insights into levels of exposure associated with toxicity in children under age five, which can inform future efforts to prevent unintended exposure to nicotine in e-liquids among children. Moreover, while the majority of cases reporting symptoms experienced nausea and vomiting, we found one severe case with oral cyanosis (bluish discoloration) and unresponsiveness.

Our study has some limitations. First, as these estimates were based on cases treated in EDs, they were likely to exclude less severe cases that did not present to EDs; conversely, fatal cases that bypassed the EDs would also be excluded. Second, since NEISS data does not include product codes specific to ENDS or provide information on poisoning severity, we had to use general keywords to capture these events, which might underestimate the burden. Third, NEISS data do not provide laboratory confirmation, such as cotinine levels, of these cases, which might lead to misclassification. Nevertheless, we were able to provide nicotine volume and concentration for cases that reported this information. Although information of nicotine dose and e-liquid volume was self-reported, those who reported this information were likely to obtain such information from reading the e-liquid bottles. On the other hand, nicotine dose and concentration information might not be accurate since research has shown that nicotine concentration can be either lower or higher than what is stated on the label (Gupta et al. 2014; Mayer 2014). Fourth, ENDS poisoning cases also occur in children over age five, but were not captured in NEISS (US Consumer Product Safety Commission, 2018a, b). Lastly, estimates might not be reliable or stable for years with estimates based on less than 20 records and national estimates of less than 1200 (US Consumer Product Safety Commission, 2017a, b). Despite these limitations, this study extends previous findings by providing national estimates of the number of ED visits for poisoning events related to e-liquids in ENDS in young children in the US.

In conclusion, our study demonstrated that thousands of young children experienced ENDS-related nicotine poisoning and were treated in hospital EDs. Some of these children developed serious clinical symptoms that could be life-threatening. While federal legislation now requires special packaging for liquid nicotine containers such as child-resistant packaging, to prevent child exposure to liquid nicotine, additional efforts, such as public education, could complement the existing child-resistant packaging law to further lower the burden of unintended nicotine exposure to ENDS e-liquids in children.

## Additional file

Additional file 1: **Table S1**. Case narratives with e-liquid nicotine volume or concentration information. **Table S2**. Case narratives with reported symptoms. (DOCX 37 kb)

## Abbreviations

CI: Confidence Interval
CNPPA: Child Nicotine Poisoning Prevention Act
ED: Emergency Department
ENDS: Electronic Nicotine Delivery System
mL: Milliliters
NEISS: National Electronic Injury Surveillance System
NPDS: National Poisoning Data System
PCC: Poison Control Center
US CPSC: US Consumer Product Safety Commission
US: United States
