# Supplementary material for: National estimates of poisoning events related to liquid nicotine in young children treated in US hospital emergency departments, 2013–2017
Source: Inj Epidemiol. 2019 Apr 1;6:10. doi: 10.1186/s40621-019-0188-9 (PMC6582692; doi:10.1186/s40621-019-0188-9)
Supplement: Supplementary file 1 — Table S1. Case narratives with e-liquid nicotine volume or concentration information. Table S2. Case narratives with reported symptoms. (DOCX 37 kb) [file 40621_2019_188_MOESM1_ESM.docx]

**National Estimates of Poisoning Events Related to Liquid Nicotine in Young Children Treated in US Hospital Emergency Departments, 2013-2017**

Joanne T. Chang, PhD, MPH (Joanne.Chang@fda.hhs.gov)

Baoguang Wang, MD, DrPH (Baoguang.Wang@fda.hhs.gov)

Cindy M. Chang, PhD, MPH (Cindy.Chang@fda.hhs.gov)

Bridget K. Ambrose, PhD, MPH (Bridget.Ambrose@fda.hhs.gov)

Center for Tobacco Products, US Food and Drug Administration, 11785 Beltsville Road, Beltsville, MD. 20705.

Corresponding author: Joanne T. Chang, US Food and Drug Administration, Center for Tobacco Products, Office of Science, Document Control Center, 11785 Beltsville Road, Beltsville, MD. 20705. Phone: 240-402-9937; Fax: 240-276-3655; Email: [joanne.chang@fda.hhs.gov](mailto:joanne.chang@fda.hhs.gov)

Abstract: 294; Text: 1403; Figure/Table: 5; References: 13; Pages: 16

**Disclaimer**: This publication represents the views of the authors and does not represent FDA/CTP position or policy.

**Supplemetary Table 1. Case narratives with e-liquid nicotine volume or concentration information**

| **Case** | **Narrative** |
| --- | --- |
| 1 | 2YOM POISONING- SWALLOWED APPROX 4OZ LIQUID VAPORIZER |
| 2 | 18 MOM ACCIDENTAL INGESTION OF VAPE JUICE CONTAINING 12 MG/ML OF NICOTINE. DX: INGESTION TOBACCO. |
| 3 | 2YOF DRANK E CIG VAPOR LIQUID, ~ 5ML; DX POISONING TOBACCO |
| 4 | 22MOM INGESTED BOTTLE OF E-CIGARETTE VAPE OIL CONTAINING NICOTENE, AT HOME, EMESIS, SLEEPY DX INGESTION / |
| 5 | 20 MOF FOUND WITH SPILLED BOTTLE OF VAPE JUICE, SMELLED ON BREATH, VOMITED X2. DX NICOTINE INGESTION |
| 6 | 3YOM POISONING- DRANK BOTTLE OF E CIGARETTE VAPOR/LIQUID |
| 7 | 1YOM AT HOME FOUND WITH A BOTTLE OF VAPORIZER SOLUTION HANDS IN MOUTH/ DX INGESTION OF VAPORIZER SOLUTION |
| 8 | 3YOM WAS FOUND WITH A 30ML BOTTLE OF '***' ELECTRONIC CIGARETTELIQUID, 25ML OF THE LIQUID WAS MISSING; ACCIDENTAL DRUG INGESTION |
| 9 | 2 YOF INGESTED 3-4ML'S OF LIQUID NICOTINE APPROX 100 MG. DX: ACCIDENTAL OVERDOSE. |
| 10 | 23 MOF FOUND WITH VAPE BOTTLE, DRANK SMALL AMOUNT, VOMITED AFTER INGESTION DX PEDIATRIC INGESTION SUSPECT NON-TOXIC |
| 11 | 22 MO FEMALE SWALLOWED BOTTLE OF "VAPE OIL". DX- INGESTION SUBSTANCE |
| 12 | 19MOF INGESTED NICOTINE E-CIG JUICE, ~0.5OZ; DX DRUG INGESTION |
| 13 | 12MOF FOUND SUCKING FROM A BOTTLE OF FAT VAPOR LIQUID NICOTENE- POISONING |
| 14 | 21MM FD A BOTTLE OF E-CIG JUICE IN A BOX&WALKED INTO THE KITCHEN DRINKING FROM THE BOTTLE, MOM CONTACTED PCC&DIR'D TO ER>>ACC ING. |
| 15 | 2YOM FND W BOTTLE DROPPER IN HIS MOUTH SUST WAS “***** ****” FOR E CIGARETTES DX POSSIBLE INGESTION |
| 16 | PT DRANK 3-4 CC OF 12MG OF LIQUID BLEND THAT GOES IN E CIGARETTES. INGESTION FOREIGN SUBSTANCE 2YOM |
| 17 | DX POISON INGESTI (E-CIGARRETE LIQUID): 2YOM DRANK ~3ML OF E-CIGARETTE FLUID, MOM STS PT HAD 1 EPS OF "SHAKING" X 1 MIN |
| 18 | E-CIGARETTE NICOTINE VAPOR INGESTION: 2YOF INGESTED E-VAPOR LIQUID THATCONTAINS NICOTINE, DRANK 1/2 BOTTLE (3MG) PEACH ***** |
| 19 | 3YOM INGESTED AUNT'S E-CIGARETTE LIQUID FROM BOTTLE OF STRAWBERRY DAQUIRI VAPOR & BLUEBERRY VAPOR,MOM UNSURE HOW FULL BOTTLES WERE;INGESTION |
| 20 | 2YOF FOUND PLAYING W/BOTTLE OF E-CIGG FLUID. POISON CONTROL CALLED DX: POSSIBLE INGESTION (NO SUBSTANCE AROUND MOUTH,NO SMELL OF LIQUID) |
| 21 | POISIONNG 3YOF POSSIBLE INGESTION 1/2ML VAPE OIL FOR E-CIG AT HOME DX: POSSIBLE INGESTION |
| 22 | POISIOING 3YOF POSSIBLE INGESTION ON CHEERY E-CIG LIQUID PARENTSFOUND OPEN BOTTLE UNK INGESTION AT HOME ACCIDENT INGESTION |
| 23 | POISIONING 17MOM CHILD DRANK <1 OZ OF VAPE JUICEDX: INGESTION |
| 24 | 20 MO M MAY HAVE DRANK 1/2 OF 15ML BOTTLE OF LIQUID NICOTINE. DX: ACCIDENTAL INGESTION OF TOXIC SUBSTANCE. |
| 25 | 9 M O M FOUND BY MOM WITH E CIGARETTE "DRIP E LIQUID 12MG ****" IN HIS MOUTH AND LIQUID ON HIS LIPS |
| 26 | 2YR M INGESTED E-CIGARETTE FLUID FROM BOTTLE; DX POISONING |
| 27 | 3YOM DRANK APPROX 5ML SMOOTH TOBACCO VAPOR FROM BOTTLE ON SHELF IN MOMS ROOM; DX ACCIDENTAL DRUG INGESTION |
| 28 | 19 MO F WAS CHEWING ON BOTTLE CAP OF 1.8MG LIQUID NICOTINE FOR E-CIGARE TTE. LIQUID ON LIPS. BOTTLE WAS ON BACK OF TV STAND. CRYING; STABLE D/C |
| 29 | 19 MOF NICOTINE FLUID INGESTION, FOUND BOTTLE OF LIQUID NICOTINE USED T FOR E-CIGARETTES. DX: INGESTION. |
| 30 | DX INGEST OF VAPOR FLAVOR FLUID 19MOM PATIENT BROUGHT IN BY PARENTS AFTER POSS INGESTION OF VAPOR FLAOR LIQUID CONTAIN 15ML FLUID |
| 31 | 3YR F INGESTED E CIG LIGUID FROM BOTTLE; DX POISONING |
| 32 | DX INGESTION OF VAPORIZER E-CIG JUICE 14MOM MOP FOUND A NICOTINE VAPOR LIQUID BOTTLE NEAR PLAY AREA PC WAS CONTACTED |
| 33 | 23MOM INGESTED VAPOR LIQUID FOR E CIGARETTES FROM BOTTLE; DX POISONING |
| 34 | 2 YOM FATHER SMOKES E CIGARETTES PT INGESTED SMALL AMOUNT 1-2 ML NICOTIE VAPOR GEL 1 EMESIS DX NICOTINE INGESTION |
| 35 | 23MOF-ACCIDENTAL INGESTION OF 5 ML OF LIQUID FROM AN E CIGARETTE-@ HOME |
| 36 | 3YOF DRANK 3/4 BOTTLE LIQUID NICOTINE FOR AN E-CIGARETTES FOUND IN UNLOCKED DRAWER AT HOME; NICOTINE INGESTION |
| 37 | 2YM ACC TOOK A SIP OF E-CIG LIQUID CONTAINS NICOTINE 1HR PTA NOW WITH VOMITING, MOM LEAVES CONTAINER ON COUNTER&GOT HOLD OF IT>>DRUG ING. |
| 38 | 2YR M INGESTED 1.2ML OF NICOTINE FLUID FOR E-CIGARETTE, DAD FOUND PT WITH IT;DX POISONING |
| 39 | 8MO M INGESTED NICOTINE FLUID FOR E-CIG, MOM FOUND PT WITH BOTTLE IN MOUTH;DX POISONING |
| 40 | 16MMO F SWALLOWED LIQUID NICOTINE, FOUND WITH BOTTLE;DX POISONING |
| 41 | 3 YR F INGESTED 1-1.5 TSP FLAVORED E CIGARETTE LIQUID. ALSO 3 PIECES OF NICOTINE GUM |
| 42 | 4YOM PUT 3 DROPS OF "***" A LIQUID NICOTINE/ TOBACCO ON TONGUE, MOM CAL LED POISON CONTROL AND BROUGHT TO ER; POISONING |
| 43 | 23MOF INGESTED 24MGS OF LIQUID NICOTENE POISONING |
| 44 | 21MONTH OLD FEMALE GOT INTO SOME LIQUID NICOTINE 1-2CC OF LIQUID DX ACCIDENTAL NICOTINE INGESTION NON TOXIC POSIONING |
| 45 | 13 MOF DRANK 5 CC VAPORIZER LIQUID DX: ACCIDENTAL INGESTION |
| 46 | PT INGESTED 18% LIQUOD NICOTINE FROM UNCLE'S VAPOR CIGARETTE. INGESTION OF FOREIGN SUBSTANCE. 15 MOF* |

Note: ***certain texts excised due to sensitive information

**Supplementary Table 2. Case narratives with reported symptoms**

| **Case** | **Narratives** |
| --- | --- |
| 1 | 22MOM INGESTED BOTTLE OF E-CIGARETTE VAPE OIL CONTAINING NICOTENE, AT HOME, EMESIS, SLEEPY DX INGESTION / |
| 2 | 23 MOF FOUND WITH VAPE BOTTLE, DRANK SMALL AMOUNT, VOMITED AFTER INGESTION DX PEDIATRIC INGESTION SUSPECT NON-TOXIC |
| 3 | 2 YO M PER MOM SHE FOUND PT DRINKING HER E-CIGARETTE JUICE, COUGHING DX INGESTION TOXIC TOBACCO AND NICOTINE |
| 4 | 16MOM GOT HOLD OF RELATIVES E CIGARETTE VIAL & INGESTED DOMEOF THE LIQUID PARENTS GRABBED HIM QUICKLY HE VOMITED SHORTLLY AFTR D NICOTINE ING |
| 5 | 2 YOM DRANK E LIQUID FOR INHALED VAPORIZER, THREW UP 1 TIME, CALLED POISON CONTROL DX INGESTION E CIGARETTE LIQUID |
| 6 | 19 MO F WAS CHEWING ON BOTTLE CAP OF 1.8MG LIQUID NICOTINE FOR E-CIGARETTE. LIQUID ON LIPS. BOTTLE WAS ON BACK OF TV STAND. CRYING; STABLE D/C |
| 7 | 2 YOM FATHER SMOKES E CIGARETTES PT INGESTED SMALL AMOUNT 1-2 ML NICOTIE VAPOR GEL 1 EMESIS DX NICOTINE INGESTION |
| 8 | 2YOF POSSIBLY INGESTED E-CIGARETTE OIL AND EXPOSURE TO EYE,EYE REDNESS; LEFT WITHOUT BEING SEEN |
| 9 | 1- MOF MOTHER FOUND PT WITH E CIGARETTE IN MOUTH INGESTED NICOTINE WAS UNRESPONSIVE/APNIC HAD ORAL CYANOSIS DX ACCIDENTAL INGESTION OF TOXIN |
| 10 | DX LWOBD: INGESTION/VOMITING: 13MOF INGESTION OF TOBACCO OIL IN VAPOR CIGARRETTES. |
| 11 | 2YM OPENED ONE OF HIS MOM BF'S E-CIGARETTES & DRANK THE LIQUID INSIDE, BEEN VOMITING, TRYING TO CLEAN HIS MOUTH>>SUBS ING. |
